# Active syndromic surveillance of COVID-19 in Israel

**DOI:** 10.1038/s41598-021-03977-3

**Published:** 2021-12-27

**Authors:** Elad Yom-Tov

**Affiliations:** 1 Microsoft Research, Alan Turing 3, Hertzliya, 4672415 Israel; 2 Faculty of Industrial Engineering and Management, Technion, Haifa, 3200000 Israel

**Keywords:** Respiratory tract diseases, Computer science

## Abstract

Syndromic surveillance systems monitor disease indicators to detect emergence of diseases and track their progression. Here, we report on a rapidly deployed active syndromic surveillance system for tracking COVID-19 in Israel. The system was a novel combination of active and passive components: Ads were shown to people searching for COVID-19 symptoms on the Google search engine. Those who clicked on the ads were referred to a chat bot which helped them decide whether they needed urgent medical care. Through its conversion optimization mechanism, the ad system was guided to focus on those people who required such care. Over 6 months, the ads were shown approximately 214,000 times and clicked on 12,000 times, and 722 people were informed they needed urgent care. Click rates on ads and the fraction of people deemed to require urgent care were correlated with the hospitalization rate ($R^2=0.54$ and $R^2=0.50$, respectively) with a lead time of 9 days. Males and younger people were more likely to use the system, and younger people were more likely to be determined to require urgent care (slope: $- \,0.009$, $P=0.01$). Thus, the system can assist in predicting case numbers and hospital load at a significant lead time and, simultaneously, help people determine if they need medical care.

## Introduction

Syndromic surveillance is the real-time or near real-time monitoring of disease indicators to detect disease outbreaks earlier than would be possible with traditional methods and to track their progression over time^1^. Passive syndromic surveillance can be achieved by medical records analysis^2,3^, participatory surveillance systems^4^, sentinel networks^5^, and internet data^6^. These methods differ in the size of their cohort, the amount of data collected from each person, and whether the data are collected continuously or only when people experience a medical event. They are mostly passive in that they rely on data that are continuously collected from a predefined cohort and that focus on well-known signals.

Internet data in general and search engine queries in particular have come to the forefront of research into syndromic surveillance, especially for tracking respiratory diseases such as influenza-like illness (ILI)^6–8^, norovirus^9^, and respiratory syncytial virus (RSV)^10^.

The application of internet-based syndromic surveillance has, to date, been limited. Several notable examples include the detection of food-borne illnesses through online searches^11^ and HealthMap’s tracking of the 2009 H1N1 influena epidemic^12^. During the recent COVID19 pandemic, Public Health England incorporated reports based on Google search data into its weekly reports (https://www.gov.uk/government/news/weekly-covid-19-surveillance-report-published).

The added value of internet data for tracking the above-mentioned conditions stems from the fact that most people with, for example, ILI, will not seek medical care but many will search about the condition or mention it in social media postings^13^.

One of the major disadvantages of passive internet-based syndromic surveillance systems is that data are often available without context and are often not medically validated. For example, the fact that a person queries for influenza treatments does not necessarily mean they are suffering from the disease. Thus, it is necessary to filter these data or use them as input to models which are tuned with data from past outbreaks for which both symptom rates and prevalence are known retrospectively.

Another drawback of internet-based syndromic surveillance systems is data access. In most cases, companies collecting internet data tightly control it for privacy reasons. Therefore, access is possible only in aggregate (e.g., Google Trends, www.trends.google.com) or in collaboration with these companies. Interestingly, the COVID-19 pandemic led Google^14^ and Bing^15^ to release more granular search engine data pertaining to the pandemic.

An alternative to passive syndromic surveillance systems are systems which actively collect syndromic data through, for example, online advertising. Search advertising allows commercial advertisers to show their ads to users who query a search engine for specific terms. The ads comprise of both text and imagery. Users who click on the ads are directed to the advertiser’s website. Users’ interactions with the ads are available in aggregate to advertisers as a way to understand the performance of their ads campaign and to improve it. Advertisers usually pay when users click on the ads. Importantly, in the context of health advertising, ad systems usually limit the terms which can be targeted by advertisers. For example, targeting pharmaceutical drugs usually requires special approval by ad-system administrators.

A novel use of advertising is syndromic surveillance, which can enable health organizations to overcome many of the challenges highlighted above, including data access. For example, Eysenbach^16^ demonstrated that the percentage of flu-related ads which were clicked by users (also known as the clickthrough rate, CTR) searching for flu-related terms was well correlated with the number of influenza cases. Ad systems can be trained to focus on specific users, based on their location, demographics, and past interests, using a feedback mechanism known as conversion optimization. This feature of the ad systems was designed to help advertisers focus on users who will purchase a product, not just click on ads. In the context of health, conversion optimization was previously used to identify people who were likely to suffer from one of three types of cancer^17^ and help them reach medical care earlier. In that work, ads were shown to people who were looking to self-diagnose one of three solid-tumor cancers. People who clicked on the ads were shown a clinically validated questionnaire whose output was used as the feedback signal to the advertising system. Responses to the questionnaire which were indicative of possible cancer were treated as positive feedback. Over time, the advertising system learned to focus on people who were likely to have questionnaire responses indicative of possible cancer, such that around 12% of the people who clicked on the ads had such questionnaire responses (from a baseline rate of under 1%).

COVID-19 was first reported in Israel in late February 2020^18^. By the end of 2020, over 425 thousand cases and 3371 deaths were reported. Here, we describe an active syndromic surveillance system for COVID-19 which builds on the above-mentioned work and was operational for approximately 6 months from April 2020. We report on the development and running of an online advertising campaign which simultaneously assisted in city-level syndromic surveillance and helped people who were experiencing relevant symptoms decide if they should seek medical care.

## Methods

### Overview

We advertised to people in Israel who made COVID-19-related symptom queries through the Google Ads platform. People who clicked on the ads were referred to the Microsoft Healthcare Bot. The interaction with the bot helped people understand the severity of their symptoms vis-à-vis their demographics and underlying medical conditions. The COVID19 instance of the Bot was created and managed by Israel’s second-largest hospital.

The advertising system was set to maximize the number of conversions. People who “converted” were defined as those people whose responses to the Healthcare Bot indicated that they were in need of urgent medical care.

Note that the algorithm used by the Healthcare Bot was independent of the advertising system. Therefore, the only way that conversion rates could be increased by the advertising system is by identifying people who were at higher likelihood for hospitalization according to the information that the ads system had about its users.

Analysis of user interactions with the ads and the Healthcare Bot at both the country and city levels helped predict COVID-19 case numbers and hospitalizations.

### Search advertising campaign

Search advertising campaign1s consist of three main elements: keywords that, if used by a searcher, will trigger the appearance of an ad; the text of the ads; and the landing page, which is the page that a user will be referred to by clicking on an ad.

Ads were shown on the Google search engine to people in Israel. Google Adwords specifies that a user’s physical location is determined by IP address or by device location (see https://support.google.com/google-ads/answer/2453995). The ads were shown in response to queries containing one of the following terms (in Hebrew): dry cough, fever, stuffy nose, difficulty breathing, diarrhea, tiredness, headache, nausea, throat pain, or vomiting. The list of symptoms was based on the United Kingdom National Health Service’s first few hundred (FF100) survey on COVID-19^19^.

The ads comprised a short (one sentence) title and a 1–3 sentence body. The ads are shown in Table 1. In practice, Google forbade ads which mentioned COVID-19, and therefore two ads were not shown by the ad system.

The campaign was run in Hebrew.

Once the elements of the campaign are decided upon (e.g., the text of the ads is prepared) setting up the campaign requires 1–2 h.

Google AdWords provides aggregated information on the results of the ad campaign, including measures of the campaign (number of impressions, clicks, and conversions) over time, stratified by city (e.g., Haifa, Jerusalem, etc.), gender, and age group (18–24, 25–34, 35–44, 45–54, 55–64, and 65 and over). We denote the number of clicks out of all impressions as the clickthrough rate (CTR). The rate of conversions out of all clicks is denoted by the conversion rate (ConvR). Aggregated information can be downloaded at any time, and its processing, to predict, e.g., future hospitalization rates, can occur in near real-time.

**Table 1:** Advertisement text.

Num	Ad title	Ad body	Disallowed
1	Are you suffering from <keyword>?	Ichilov’s corona bot will help you decide if you need medical attention	No
2	Are you worried you may be ill with COVID-19?	Ichilov’s corona bot will help you decide if you need medical attention	Yes
3	Are you running a fever?	Ichilov’s corona bot will help you decide if you need medical attention	No
4	Do you have a severe cough?	Cory, Ichilov’s bot will help you decide if you need medical attention	No
5	Do you know someone who is ill with COVID-19? Are you feeling ill?	Ichilov’s cory will help you decide if you need medical attention	Yes

The token <keyword> refers to the keyword which caused the ad to be shown. Ichilov is the colloquial name of the Tel Aviv Sourasky Medical Center. Disallowed ads were not permitted by the ad system.

### Microsoft health bot

People who clicked on the campaign ads were referred to an instance of the Microsoft Healthcare Bot (https://www.microsoft.com/en-us/research/project/health-bot/) which was installed by the Tel Aviv Sourasky Medical Center, Israel’s second largest hospital. The bot was configured by medical professionals to ask people about their demographics, underlying health conditions, and the symptoms they were experiencing. At the end of the interaction, the bot provided people with one of three outcomes: stay at home, call the family physician, or go to an emergency room. The latter was used as a feedback signal to the conversion optimization mechanism.

### Ground truth case data

We retrospectively compared data from the advertising campaign to the Israeli Ministry of Health’s COVID-19 data repository (https://data.gov.il/dataset/covid-19). Specifically, our data was compared to the COVID-19 by area dataset, which provides information on the number of new COVID-19 cases per 100,000 people in each city on each day and the number of hospitalizations at these temporal and spatial resolutions. We refer to these as the case rate and hospitalization rate, respectively.

We note that case counts and hospitalizations are known to be noisy proxies for disease incidence, affected by policy, resources, and test accuracy. However, at the time of writing, these are the most comprehensive publicly available city-level data on COVID-19 in Israel.

### Demographic data

We collected city-level demographic data from two official sources: Total population and the percentage of Arab citizens in a city from the Israeli Statistic Bureau^20^ and the median monthly income (2011), percentage of people earning less than the minimum wage and Gini index from the Israel National Insurance Institute^21^. These were used to evaluate differences in the demographics of people who utilized the campaign.

### Data analysis

The correlation between the advertising-system measures and the pandemic indicators, taking into account the share of keywords for which the campaign ads were shown, was estimated through a linear model operating at daily granularity. The independent parameters of the model were either CTR or ConvR, expenditure per day, and the share of search impressions that day. The dependent parameters of the model were the case rates and hospitalization rates. Since it is hypothesized that campaign data may predict future indicators of the pandemic, the model is estimated at different lags between the data of the advertising system and those of the pandemic indicators, where a negative lag of *k* days means that data from the advertising system is correlated with pandemic data taken *k* days later. Models were estimated for lags ranging from − 14 to + 14 days.

## Results

### The advertising campaign

The advertising campaign was run between April 9th and October 12th, 2020. On average, the display of the ads cost US $16 per day. Campaign ads were shown approximately 214,000 times and clicked 11,924 times. During this time period, 722 people completed the interaction with the bot and were advised to reach an emergency room.

**Figure 1:**
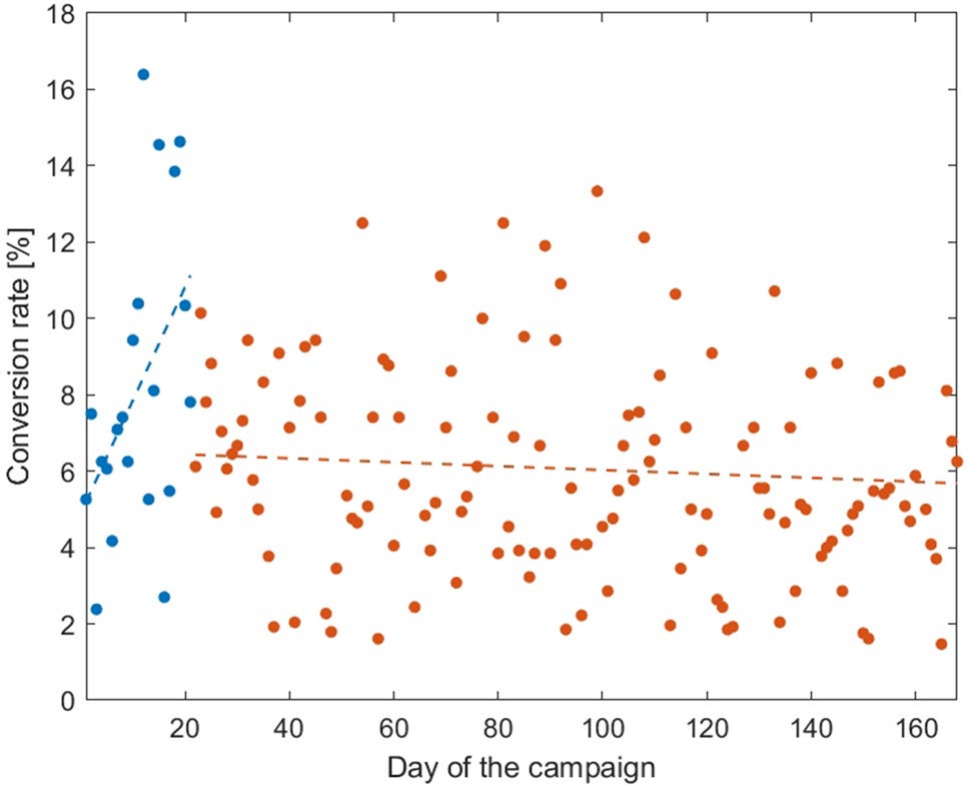
Conversion rate per day over the duration of the advertising campaign. The first 21 days of the campaign are colored in blue and the rest in brown. Conversion rate rose during the first 21 days of the campaign (as indicated by the blue regression line) and then plateaued (as shown by the brown regression line).

Through the conversion optimization mechanism, the advertising system was trained to focus on people who it predicted would require referral to urgent medical care. Specifically, we provided a conversion signal from people who received feedback from the Health Bot referring them to urgent care. The average conversion rate per day over the entire campaign was 5.8%. This conversion rate was statistically significantly correlated with CTR ($r=0.20$, $P=0.007$). As Fig. 1 shows, the conversion rate per day rose during the first 21 days of the campaign ($r=0.47$, $P=0.033$), after which it plateaued and was not statistically significantly correlated with the day of the campaign ($r=-\, 0.09$, $P>0.05$).

The ad system provides limited information on the performance of the campaign among different populations. An analysis of this information reveals differences in responses to the ad by gender, age, and location. Specifically, CTR among males (6.4%) was higher than that of females (5.6%) ($\chi ^2$ test, $P<10^{-5}$). Similarly, ConvR of males (6.5%) was higher than that of females (5.0%) ($\chi ^2$ test, $P=0.002$).

People aged 18–24 had the highest CTR (8.8%), compared to approximately 5.0% for other age groups ($\chi ^2$ test, $P<10^{-5}$). However, ConvR was slightly higher in ages up to 44 (approximately 7.0%), compared to around 4.0% for older people ($\chi ^2$ test, $P<10^{-5}$).

A linear regression model in which the independent attributes are gender and age and the dependent variable is CTR is not statistically significant. However, the same model of ConvR reaches $R^2=0.58$ ($P=0.020$). In this model, only age is statistically significantly correlated (slope: $-\, 0.009$, $P=0.015$).

City-level models of the number of impressions, CTR and ConvR as a function of the different city’s demographic indicators (see “Demographic data” section) were built to test the economic indicators associated with use of the campaign ($n=54$). A model of the number of impressions reached an $R^2$ of 0.60 with the only statistically significant variable being population (slope: 0.02, $P=10^{-9}$). A model of CTR reached $R^2=0.35$, with the fraction of Arab citizens being statistically significant (slope: 0.01, $P=0.015$). Finally, the model of ConvR was not statistically significant.

The keywords with the highest CTR were difficulty breathing (10.2%), stuffy nose (5.2%), and dry cough (4.9%). Keywords with the highest ConvR were difficulty breathing (ConvR: 7.9%), throat pain (ConvR: 3.3%), and dry cough (ConvR: 2.7%).

Most of the campaign’s ads were shown to mobile platform users (96%), followed by computers (3%), and tablets (1%). However, conversions were most likely on computers (8%) and less so on mobile (6%) and tablets (2%).

### Correlation of advertising-campaign and pandemic indicators

**Figure 2:**
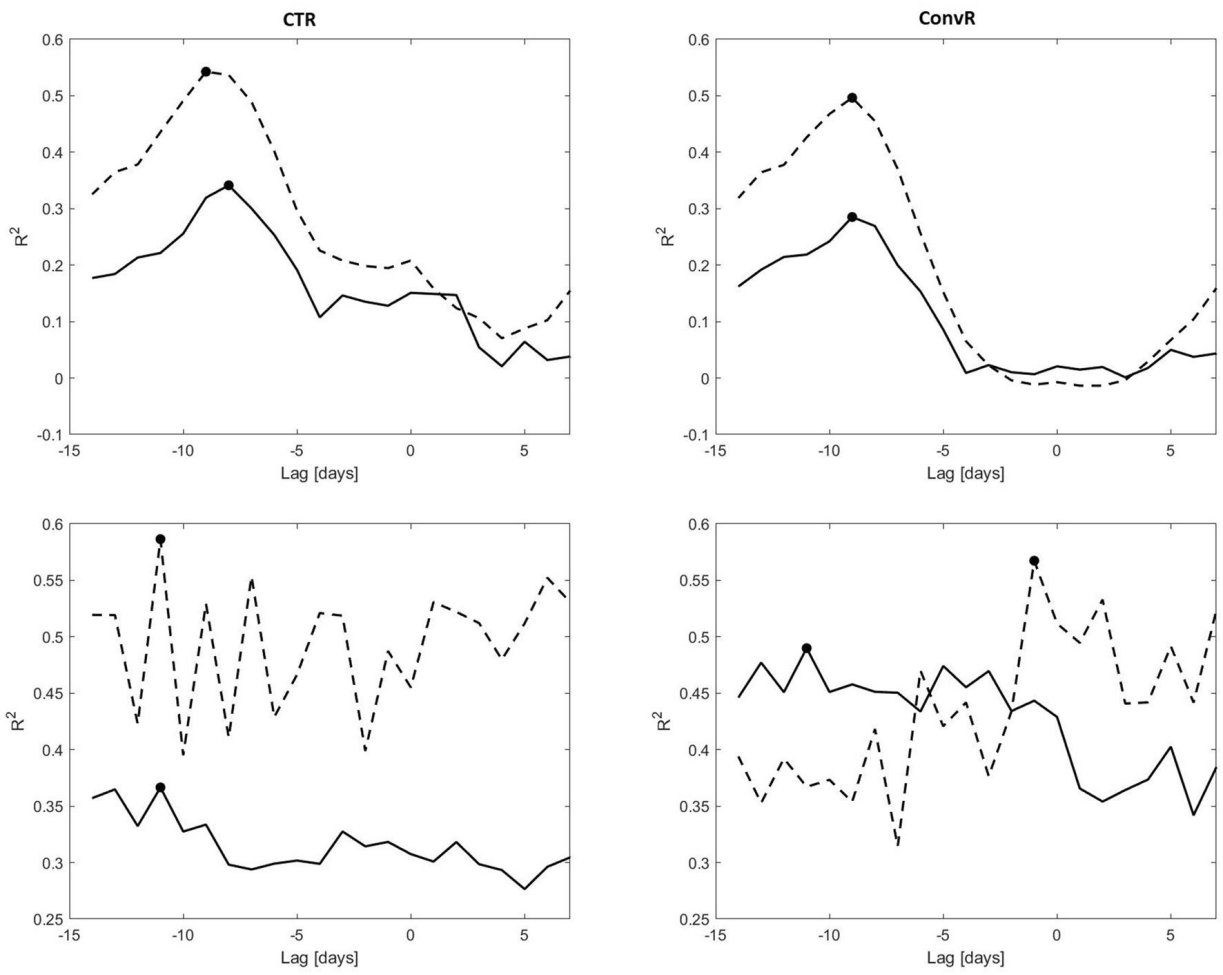
Coefficient of determination ($R^2$) as a function of lag for different parameters of the advertising system and indications of the pandemic. The top figures show data for the entire country, while the bottom are at a city level. The left column shows the correlation with CTR and the right column with ConvR. Bold lines denote case rates and dotted lines hospitalization rates. The dots show the highest correlation for each curve. Negative lag indicates that data from the advertising system leads that of the pandemic. Note that vertical axes have different values for country- and city-level graphs.

The coefficients of determination ($R^2$) between advertising-campaign indicators (CTR and ConvR) and pandemic indicators (case rate and hospitalization rate ) as a function of the lag between the two, for both country- and city-level data, are shown in Fig. 2. The highest value for each is given in Table 2. Note that city-level analysis was conducted for all cities which had at least 20 clicks and 10 days with non-zero conversions over the entire period.

The highest correlation is statistically significant in all cases ($P<10^{-5}$) and is highest for the correlation between CTR and hospitalizations. The best correlations are reached when data from the advertising system is 8–9 days prior to that of the pandemic indicators. Country-wide correlations are less noisy than city-level indicators, as can be expected. Nevertheless, city-level results are consistent with those of the entire country, with the exception of ConvR, which is correlated with the hospitalization rate at a lag of one day.

**Table.2:** Highest coefficient of determination ($R^2$) and its lag in days (in parenthesis), for different parameters of the advertising system and indications of the pandemic.

Pandemic indicator	Entire country		City level	
	CTR	ConvR	CTR	ConvR
Case rate	0.34 (− 8)	0.29 (− 9)	0.37 (− 11)	0.49 (− 11)
Hospitalization rate	0.54 (− 9)	0.50 (− 9)	0.59 (− 11)	0.57 (− 1)

Negative lag indicates that data from the advertising system leads that of the pandemic.

## Discussion

Many syndromic surveillance methods exist for discovering emerging disease outbreaks and for monitoring ongoing epidemics. These methods, however, often rely on known cohorts or groups of volunteers, specific disease diagnoses, or known symptom combinations. When a novel outbreak begins, adapting these systems to the new disease can be difficult and can take valuable time.

An alternative, especially when some of the people affected by the outbreak may not visit a health provider, is to field an active syndromic surveillance system as described here. Specifically, this system requires two main components: an online advertising system capable of optimizing its resources according to feedback and a questionnaire which provides the system with feedback on whether people who clicked on the ads were relevant to the tracked disease. As we showed, the questionnaire can have the added benefit of informing people whether they should seek medical treatment.

The advantage of the syndromic surveillance system described here is that it does not require people to have a verified indication of their condition, which they may not have either because they are not diagnosed yet or because they perceive their condition as not serious enough to warrant visiting a health provider.

Our findings indicate that the syndromic surveillance system learned to focus on seriously ill people within the first 21 days of its operation (see the sharp rise in conversion rate during the first 21 days shown in Fig. 1), reaching a conversion rate of almost 6%. After this initial period, conversion rates fluctuate, but this overall slope is close to zero indicating that the advertising system could not significantly increase the conversion rate.

During six months of deployment, the system was used by around 12,000 people of which 722 required urgent medical care. Although we do not have information on whether people who were urged to seek medical help did so, past work suggest that many would^17^. Future work may attempt to use geographic conversion mechanisms (e.g., a conversion which occurs if the user later appears within a healthcare facility) to directly optimize behavior change, similar to the work of Mohanty et al.^22^.

Males were more likely to use the system and were more likely to be identified as having a serious illness. Though the system was used regardless of age and gender, young people were more likely to have serious illness. We attribute both findings to those populations being less likely to seek traditional medical attention^23^ and also to the fact that younger people feel more comfortable with an online diagnostic platform^24,25^. Interestingly, the campaign was utilized regardless of economic indicators, implying that people of different social strata were similarly likely to use the system. Additionally, Arab citizens were more likely to click on the campaign ads even though they were provided in Hebrew.

The correlation of CTR and ConvR with pandemic indicators was similar, and was strongest at a lag of 8–9 days. Interestingly, the correlation was stronger with the hospitalization rate than with the number of cases. We attribute this to the implementation of the ad system’s conversion optimization mechanism which provided feedback to the ad system to focus on the most severe cases of COVID-19, according to users’ interactions with the Healthcare Bot. Thus, our results suggest an ability to focus on specific segments of the population, for example, those who are severely ill, helping them to reach treatment and assisting health authorities in predicting hospital loads at a significant lead time.

The active syndromic surveillance described herein requires knowing that a particular pathogen is circulating in the population, that its symptoms can be recognized by people, and that there exists a clinical questionnaire that can give an indication that a person is ill with the relevant condition. The first limitation means that, unless a very general symptom questionnaire is used, the active syndromic surveillance system can only be implemented once a disease has been identified and a questionnaire for it is designed. The second limitation means that only diseases with external physical symptoms can be addressed. Nevertheless, it may be possible to create a general purpose questionnaire that identifies serious or unusual symptoms, thus creating a system which detects novel diseases appearing in the population, without predefining the specific illness that needs to be tracked, overcoming the first of the above-mentioned limitations.

Our campaign was seen by up to approximately 2% of the population (some people may have seen the ads multiple times). A more wide-reaching campaign could have been seen by a larger portion of the population, thereby having a larger influence on the public’s behavior. A wider reach would have the benefit of helping more people reach a useful decision, but it might also reduce the lead time between measures of the ad system (CTR and ConvR) and pandemic indicators (cases and hospitalizations) as it would cause people to reach healthcare systems earlier. Notwithstanding this consideration, while search engines are by far the most widely used method for health-related searches^26^, not all segments of the population use the internet to the same extent. Therefore, active syndromic surveillance should not be the only method used to track a pandemic. Nevertheless, we believe that our system offers a novel and useful complement to other syndromic surveillance methods.
